# Xuezhikang contributes to greater triglyceride reduction than simvastatin in hypertriglyceridemia rats by up-regulating apolipoprotein A5 via the PPARα signaling pathway

**DOI:** 10.1371/journal.pone.0184949

**Published:** 2017-09-21

**Authors:** Shui-ping Zhao, Rong Li, Wen Dai, Bi-lian Yu, Lu-zhu Chen, Xian-sheng Huang

**Affiliations:** 1 Department of Cardiovascular Medicine, The Second Xiangya Hospital, Central South University, Changsha, Hunan, China; 2 Department of Stomatology, The Second Xiangya Hospital, Central South University, Changsha, Hunan, China; University of Kansas Medical Center, UNITED STATES

## Abstract

Xuezhikang (XZK), an extract of Chinese red yeast rice, is recommended as an optimal choice for patients with coronary heart disease (CHD) with markedly elevated triglyceride (TG) levels. This study was designed to compare the hypotriglyceridemic effects between XZK and simvastatin. The role of apolipoprotein A5 (apoA5), a key regulator of TG metabolism and a target gene of peroxisome proliferator-activated receptor α (PPARα), was to be identified in XZK-related hypotriglyceridemic actions. For these goals, hypertriglyceridemia of rats was induced by a high-fructose diet. In order to investigate the hypotriglyceridemic effects of XZK and simvastatin on these animals based on an equivalent low-density lipoprotein cholesterol (LDL-C) lowering power, we titrated their doses (XZK 80 mg/kg/d *versus* simvastatin 1 mg/kg/d) according to plasma LDL-C reduction of rats. Similarly, we titrated the target doses of the two agents (XZK 500 μg/ml *versus* simvastatin 10 μM) according to hepatocyte LDL receptor expressions, and then compared the effects of the two agents on TG and apoA5 of hepatocytes *in vitro*. Our results showed that XZK (80 mg/kg/d) had higher hypotriglyceridemic performance than simvastatin (1 mg/kg/d) on these animals albeit their equivalent LDL-C lowering power. Higher plasma apoA5 levels and hepatic apoA5 expressions were observed in rats treated with XZK (80 mg/kg/d) than simvastatin (1 mg/kg/d). Further, XZK (80 mg/kg/d) contributed to higher hepatic PPARα expressions of rats than simvastatin (1 mg/kg/d). Although the two agents led to an equivalent up-regulation of LDL receptors of hepatocytes, more TG reduction and apoA5 elevation were detected in hepatocytes treated with XZK (500 μg/ml) than simvastatin (10 μM). However, PPARα knockdown eliminated the above effects of XZK on hepatocytes. Therefore, our study indicates that XZK has greater hypotriglyceridemic performance than simvastatin in the setting of an equivalent LDL-C lowering power, which is attributed to more apoA5 up-regulation by this agent via the PPARα signaling pathway.

## Introduction

Lipid disorders are well-acknowledged as important risks for the pathogenesis of coronary heart disease (CHD). Although elevated low-density lipoprotein cholesterol (LDL-C) represents a major contributor for CHD [1], it is frequently observed that plasma triglyceride (TG) levels still remain high in some CHD patients despite their satisfactory reduction of LDL-C by statins (also known as HMG-CoA reductase inhibitors), a widely-prescribed class of lipid-lowering medications that reduce atherosclerotic cardiovascular risk primarily via LDL-C reduction [1]. However, this remaining TG elevation of CHD patients after statin therapy has been demonstrated to increase their residual cardiovascular risks [2]. In China, most CHD patients choose Xuezhikang (XZK), an extract of Chinese red yeast rice, containing a family of natural statins [3]. Interestingly, according to the definition of low-intensity statin therapy as lowering LDL-C by less than 30% with a daily-dose statin [1], XZK is categorized as a low-intensity statin because its average daily dose (1200 mg/d) results in an approximately 27% reduction of LDL-C [4]. Despite its modest performance in LDL-C reduction, XZK remains popular among Chinese CHD patients that is, to a large extent, attributed to its excellent performance in TG reduction [3, 4]. Compared to Western Caucasian CHD population [5], a hallmark feature of lipid disorders in Chinese CHD patients is that they are characterized with modestly increased LDL-C but often markedly elevated TG [6]. Given that XZK has a higher performance in TG reduction than a low-intensity statin (such as simvastatin 10 mg/day), it is reasonable to recommend this agent as an optimal choice for these CHD patients. To date, however, no relevant studies have been designed to compare their hypotriglyceridemic performance between XZK and a low-intensity statin.

This study selected apolipoprotein A5 (apoA5), an important regulator of TG metabolism [7], as a potential player to compare the hypotriglyceridemic performance between XZK and simvastatin. ApoA5 was first identified as a novel member of the apolipoprotein gene superfamily simultaneously by two independent research groups in 2001 [8, 9]. The human APOA5 gene is located on chromosome 11q23, approximately 30 kb downstream from the well-known APOA1/C3/A4 gene cluster, which consists of four exons encoding a 343 amino acids and 39 kDa protein (apoA5) that is exclusively synthesized in the liver, secreted into the plasma, and extensively distributed on chylomicrons, very low-density lipoprotein (VLDL) and high-density lipoprotein (HDL). Albeit very low plasma concentrations of apoA5 (approximately 0.1% w/w of apoA1) [9], this protein has a tremendous and negative impact on plasma TG homeostasis. Knocking out the endogenous APOA5 gene in mice confers a four-fold increase of plasma TG levels [9], whereas high-expression of the human APOA5 gene in mice leads to a decrease of TG by 50–70% [10]. Moreover, human genetic studies have identified several APOA5 polymorphisms that affect plasma TG levels [11, 12]. Inherited deficiency of the APOA5 gene in humans leads to severe hypertriglyceridemia [13]. Of note, apoA5 is implicated in the hypotriglyceridemic effects of two common lipid-lowering agents, fibrates and statins [14–16]. Nevertheless, the role of apoA5 in XZK-related hypotriglyceridemic actions is still uncertain, and identifying it was the purpose of this study. Considering XZK as a multi-component polypill, including thirteen kinds of natural statins, unsaturated fatty acids, ergosterols and so on [4], it is unlikely to attribute the whole lipid-modulation benefits of this agent only to its statin components. Therefore, this study adopted tactics for an equivalent LDL-C lowering power of XZK and simvastatin, which was designed to compare the differences of their hypotriglyceridemic effects.

## Materials and methods

### Animal experiments and measurements

Male, 8-week old, Sprague-Dawley rats (n = 40, Shanghai Slac, China) were housed and maintained in specific pathogen-free conditions with a 12-h light and 12-h dark cycle. Food and water were provided *ad libitum* to the animals in standard cages. The Animal Care and Use Committee (ACUC) of Central South University (CSU) approved the study design and all experimental protocols used. All experiments were performed in accordance with the guidelines of ACUC. Adequate maneuvers were taken to minimize any pain and discomfort to these experimental animals. Food intake and body weight were recorded once a week. The adverse events (including signs of illness or mortality) of animals were monitored for three times per day, and no adverse events were observed throughout the study. Hypertriglyceridemia rat models were set up by a high-fructose diet as described previously [16]. These animals were randomized into four groups (n = 10 each group), including: (1) control group; (2) fructose group (*i*.*e*. fructose-induced hypertriglyceridemia group); (3) statin group; and (4) XZK group. For the fructose group and the two drug-treated groups, we first established the hypertriglyceridemic animal model with fructose supplied as a 10% solution in drinking water for 2 weeks. Then, the different treatments were given to these three groups as follows: (1) the fructose group continued on 10% fructose (AMRESCO, Solon, OH, USA) in drinking water for 4 weeks; (2) the two drug groups were given the 10% fructose in drinking water plus either simvastatin (1 mg/kg/d) or XZK (80 mg/kg/d) for 4 weeks. Since the primary goal of this study was to compare the TG-lowering (not the LDL-C-lowering) effects of the two agents, we titrated their doses (XZK 80 mg/kg/d versus simvastatin 1 mg/kg/d) according to their reduction of plasma LDL-C levels in animals for an equivalent LDL-C lowering power. Simvastatin (Simvastatin powder was kindly provided by the Merck China Co, Hanzhou, China) and XZK (Xuezhikang powder was kindly provided by the WBL Peking University Biotech Co, Beijing, China) were administered by daily oral gavage, using an aqueous carboxymethyl cellulose suspension vehicle (0.5% carboxymethyl cellulose plus 0.1% Tween 80 in water). After 6 weeks, all animals were sacrificed by decapitation under anesthesia using sodium pentobarbital (50 mg/kg, *i*.*p*.). Blood samples were collected at death in 5% EDTA tubes stored at −80°C until analysis. Plasma apoA5 levels were measured by an ELISA method (sc-33081, Santa Cruz). Plasma TG and total cholesterol were measured by commercially available kits (Merck-Labkit, Spain) and HDL-C was detected by a direct method (Accurex biomedicals, India) [16]. Each time, quality control sera (BioRad, USA) was run along with the unknown samples. The LDL-C was calculated via the following Friedwald equation: LDL-C (mmol/L) = [Total cholesterol–(HDL-C + TG/2.2)]. Liver samples were immediately frozen and stored at –80°C until needed. Hepatic mRNA and protein levels of apoA5 and peroxisome proliferator-activated receptor α (PPARα) were quantified by real-time quantitative polymerase chain reaction (RT-qPCR) and Western blot analysis, respectively.

### Cell experiments

Cell experiments were conducted as we described previously [16], with the following exception: Considering the fact that LDL-C (primarily existing in circulation) cannot be measured in cultured hepatocytes *in vitro* and LDL receptor (LDL-R) on hepatocytes plays a key role in statin-related LDL-C reduction [17], hepatocyte LDL-R protein expressions by Western blot analysis were used as an index to titrate the target doses of the two agent (XZK 500 μg/ml versus simvastatin 10 μg/ml) for an equivalent LDL-C lowering power (namely an equivalent up-regulation of hepatocyte LDL-R expressions) *in vitro*. Simvastatin powder (Merck China, Hanzhou, China) was dissolved in dimethyl sulfoxide (DMSO). XZK powder (WBL Peking University Biotech Co, Beijing, China) was dissolved in DMSO and subsequently the soluble liquid was vortexed. The dosage solvent of XZK was obtained by ultrasonification for 40 minutes and then filtering out the remainder of the insoluble starch and fiber using a 4.5 mm filter. The final concentrations of DMSO were maintained at 0.1% (v/v). HepG2 cells were divided into five groups: (1) control group, treated with 0.1% DMSO alone; (2) fructose group, with 100 μg/ml fructose; (3) statin group, with 10 μg/ml simvastatin and 100 μg/ml fructose; (4) XZK group, with 500 μg/ml XZK and 100 μM fructose; (5) PPARα KO (knockdown) + XZK group, with 500 μg/ml XZK and 100 μM fructose in the setting of PPARα knockdown performed with an adenovirus-based shRNA method (Genechem Biotech Co, Shanghai, China). In detail, cells were infected with adenovirus for 24h before adding XZK and fructose. After 24-hour incubations, cells were washed and cellular TG contents were measured by enzymatic reagents (Boehringer Mannheim, USA) as previously described [16]. Cellular TG results were reported as μg of cellular TG per mg cell protein. The cellular expressions of apoA5 and PPARα were determined by RT-qPCR and Western blot analysis.

### RT-qPCR analysis

APOA5 and PPARα mRNAs abundance were assessed by RT-qPCR as described previously in our another study [18]. Total RNAs were extracted from liver tissues or HepG2 cells using Trizol reagent (Invitrogen) according to manufacturer’s instructions. RNAs were reversely transcribed into cDNA using Prime Script ® RT reagent Kit (Takara). The cDNA samples were amplified in duplicate in 96-microtiter plates (Applied Biosystems). Each PCR reaction (20 μl of total volume) contained: 10 μl of SYBR Green PCR Master Mix (Applied Biosystems), 5 pmols of each primer and 1 μl of cDNA. RT-qPCR reactions were carried out in an ABI PRISM 7, 500 RT-qPCR apparatus. The thermal profile settings were 95°C for 2 min, then 40 cycles with 15 s at 95°C for denaturation and with 1min at 60°C for annealing and final extension. The dissociation curves were analyzed to check for gene-specific amplification, no non-specific products were detected. The GAPDH gene was used as the internal control. For each gene, the relative expression was calculated and shown as a fold-change using 2-ΔΔCt method, normalized to GAPDH. Gene-specific primers for RT-qPCR were designed using the Primer Express ® Software v3.0 (Life Technologies). The sequences of sense and antisense primers were shown in Table 1.

**Table 1:** Oligonucleotide sequences of primers and shRNA targeting human PPARα gene.

Gene	Source	Category	sequences
PPARα	Rat	Primers	Forward: 5´- CAA TGG CTT CAT CAC CCG AG -3´
			Reverse: 5´- ATC CCC TCC TGC AAC TTC TC -3´
PPARα	Human	Primers	Forward: 5´- ATG GTG GA CAC GGA AAG CC-3´
			Reverse: 5´- CGA TGG ATT GCG AAA TCT CTT GG -3´
APOA5	Rat	Primers	Forward: 5'- CCA ACG CAA CCT AGA TCA GC -3'
			Reverse: 5'- CCT GAG TGA ATG CAG CGA TC -3'
APOA5	Human	Primers	Forward: 5'- CCG CGA CCC TGA AAG ACA -3'
			Reverse: 5'- CAA AGC CCA AGC CTC GTC -3'
GAPDH	Rat	Primers	Forward: 5'- AAC GAC CCC TTC ATT GAC CT -3'
			Reverse: 5'- AAG ACG CCA GTA GAC TCC AC -3'
GAPDH	Human	Primers	Forward: 5'- TGT GGG CAT CAA TGG ATT TGG -3'
			Reverse: 5'- ACA CCA TGT ATT CCG GGT CAA T -3'
PPARα	Human	shRNA	5'-CCG GGT AGC GTA TGG AAA TGG GTT TCT CGA GAA ACC CAT TTC CAT ACG CTA CTT TTT-3'

### Western blot analysis

Western blot analysis was performed as described previously [16]. Briefly, 50μg of each protein sample was separated on 10% SDS-PAGE and transferred onto PVDF membrane. The membrane was incubated overnight with a primary antibody against each target protein (mouse monoclonal anti-apoA5 antibody, Santa Cruz; or rabbit monoclonal anti-PPARα antibody, Abcam; mouse monoclonal anti-LDL receptor antibody, Progen Biotechnik) at 4°C overnight. After incubation with an HRP-conjugated secondary antibody, immunoreactive bands were visualized using the enhanced chemiluminescence detection system. Data were quantified by densitometry after scanning, using the TINA software (Raytest, Germany). The results of the target protein were presented relative to GAPDH expression.

### Statistical analyses

Data were analyzed using SPSS 15.0 (SPSS Inc.) and presented as the mean ± SD unless otherwise indicated. Log transformation was made for distribution-dependent analyses. Differences between intragroup and intergroup means were analyzed by Student t-test or one-way ANOVA. Coefficients of correlation (r) were calculated by the Pearson correlation analysis. P-value <0.05 was considered statistically significant.

## Results

### XZK leads to greater plasma apoA5 elevation and TG reduction than simvastatin in the setting of an equivalent LDL-C lowering power in rats

As shown in **Table 2**, six weeks of the high-fructose diet led to a remarkable increase of plasma TG levels in fructose group (2.16 ± 0.27 mmol/L at week six versus 0.73 ± 0.17 mmol/L at baseline, P < 0.001), suggesting our fructose-induced hypertriglyceridemia rat models were successfully established. Conversely, a significant reduction of plasma apoA5 levels was observed in fructose group at week six from the baseline (68.76 ± 12.33 ng/mL at week six versus 128.09 ± 15.15 ng/mL at the baseline, P < 0.001). Remarkably, although the two treatments led to a notable reduction of plasma LDL-C levels (both P < 0.001), no significant difference of LDL-C was demonstrated between the two treatment groups at week six (1.18 ± 0.17 mmol/L in statin group versus 1.19 ± 0.18 mmol/L in XZK group, P > 0.05), which indicated the two treatments (XZK 80 mg/kg/d versus simvastatin 1 mg/kg/d) shared an equivalent LDL-C lowering power. However, more TG reduction was shown in XZK group than statin group at week six (1.18 ± 0.17 mmol/L in XZK group versus 1.52 ± 0.21 mmol/L in statin group, P < 0.05). By contrast, the two treatments induced an elevation of plasma apoA5 levels, and these up-regulative effects on apoA5 were more apparent in XZK group than statin group (106.25 ± 15.08 ng/mL in XZK group versus 89.65 ± 13.52 ng/mL in statin group, P < 0.05).

**Table 2:** Plasma LDL-C, TG, and apoA5 in rats (n = 10 each group).

		LDL-C (mmol/L)	TG (mmol/L)	ApoA5 (ng/mL)
Control	Baseline	1.28 ± 0.14	0.72 ± 0.16	127.52 ± 15.20
	Week Six	1.32 ± 0.17	0.76 ± 0.18	125.67 ± 16.54
Fructose	Baseline	1.28 ± 0.16	0.73 ± 0.17	128.09 ± 15.15
	Week Six	1.69 ± 0.25 ^1^^,^^2^	2.16 ± 0.27 ^1^^,^^2^	68.76 ± 12.33 ^1^^,^^2^
Statin	Baseline	1.29 ± 0.18	0.74 ± 0.13	126.98 ± 17.59
	Week Six	1.18 ± 0.17 ^1^^,^^2^^,^^3^	1.52 ± 0.21^1^^,^^2^^,^^3^	89.65 ± 13.52 ^1^^,^^2^^,^^3^
XZK	Baseline	1.28 ± 0.15	0.73 ± 0.15	127.79 ± 16.10
	Week Six	1.19 ± 0.18 ^1^^,^^2^^,^^3^	1.18 ± 0.17 ^1^^,^^2^^,^^3^^,^^4^	106.25 ± 15.08 ^1^^,^^2^^,^^3^^,^^4^

^1^Values at week six were significantly different from baseline (P < 0.05).

^2, 3, 4^ Values at week six were significantly different from the control, fructose, and statin groups, respectively (P < 0.05).

LDL-C, low density lipoprotein cholesterol; TG, triglyceride; ApoA5, apolipoprotein A5; XZK, Xuezhikang.

In order to investigate the relationship between apoA5 and TG, correlation analyses were conducted. As shown in **Table 3**, a significant inverse correlation was identified between the two factors in each group at the baseline or at week six. After pooling all data in each group at the baseline and week six, the inverse relationship between apoA5 and TG remained.

**Table 3:** Correlations between apoA5 and TG in rats (n = 10 each group).

	Control		Fructose		Statin		XZK	
	r	P	r	P	r	P	r	P
Baseline	− 0.657	0.015	− 0.649	0.020	− 0.675	0.011	− 0.654	< 0.017
Week Six	− 0.702	0.008	− 0.685	0.009	− 0.689	0.008	− 0.708	< 0.006
All	− 0.678	0.010	− 0.659	0.013	− 0.682	0.010	− 0.679	< 0.010

ApoA5, apolipoprotein A5; XZK, Xuezhikang; r, correlation coefficient.

### XZK upregulates hepatic expressions of apoA5 and PPARα more than simvastatin in the setting of an equivalent LDL-C lowering power in rats

**Fig 1:**
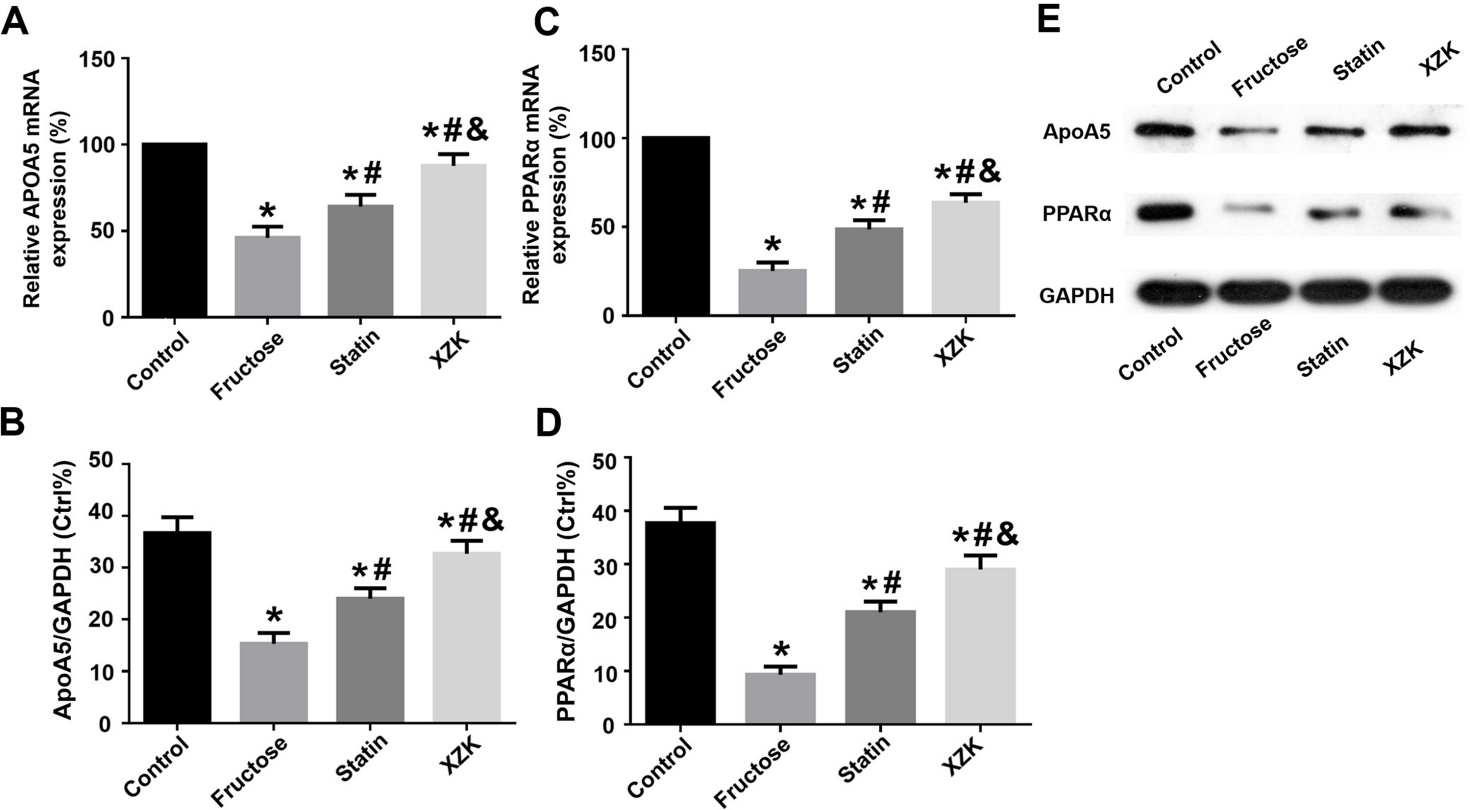
Hepatic apoA5 and PPARα mRNA and protein expressions in rats. The mRNA and protein levels of hepatic apoA5 and PPARα in rats were detected by RT-qPCR and Western blot analysis respectively: (A and B) ApoA5 mRNA and protein- Compared with control group, a significant reduction of apoA5 mRNA and protein was observed in fructose group. However, the two treatments effectively ameliorated fructose-induced down-regulation of apoA5 expressions, whereas this effect was more considerable in XZK group. (C and D) PPARα mRNA and protein- Similar findings of hepatic PPARα expressions were obtained, i.e. the two treatments attenuated fructose-induced down-regulation of hepatic PPARα expressions but this effect was more remarkable in XZK group. E ApoA5 and PPARα protein results by Western blot analysis. *^, #, &^ Values were significantly different from control, fructose, and statin groups, respectively (P < 0.05). ApoA5, apolipoprotein A5; PPARα, peroxisome proliferator-activated receptor α; XZK, Xuezhikang.

Considering APOA5 as a liver-specific factor and a target gene of PPARα [14], hepatic expressions of apoA5 and PPARα of animals were detected. As shown in **Fig 1**, hepatic expressions of apoA5 mRNA and protein were consistent with the plasma profile of this protein. Compared with control group, a significant reduction in apoA5 mRNA and protein was detected in fructose group (both P < 0.05). It was consistent with our previous findings [16] that revealed that high-fructose diet can induce hypertriglyceridemia in rats by inhibiting hepatic apoA5 expressions. However, simvastatin (1 mg/kg/d) or XZK (80 mg/kg/d) effectively ameliorated fructose-induced apoA5 down-regulation (all P < 0.05), whereas this effect was more remarkable in XZK group than statin group (P < 0.05). Thus, it indicated that XZK (80 mg/kg/d) more effectively decreased plasma TG levels of animals than simvastatin (1 mg/kg/d) despite their equivalent performance in LDL-C reduction.

Similarly, consistent findings about hepatic PPARα mRNA and protein were obtained. Compared with controls, the high-fructose diet remarkably inhibited hepatic PPARα mRNA and protein (both P < 0.05). Nevertheless, the two treatments apparently attenuated fructose-induced PPARα down-regulation (all P < 0.05), and this effect was more considerable in XZK group than statin group (P < 0.05). Therefore, it implied that XZK (80 mg/kg/d) contributed to more PPARα up-regulation of these animals than simvastatin (1 mg/kg/d).

### XZK contributes to greater apoA5 elevation and TG reduction than simvastatin in hepatocytes *in vitro* via the PPARα pathway

**Fig 2:**
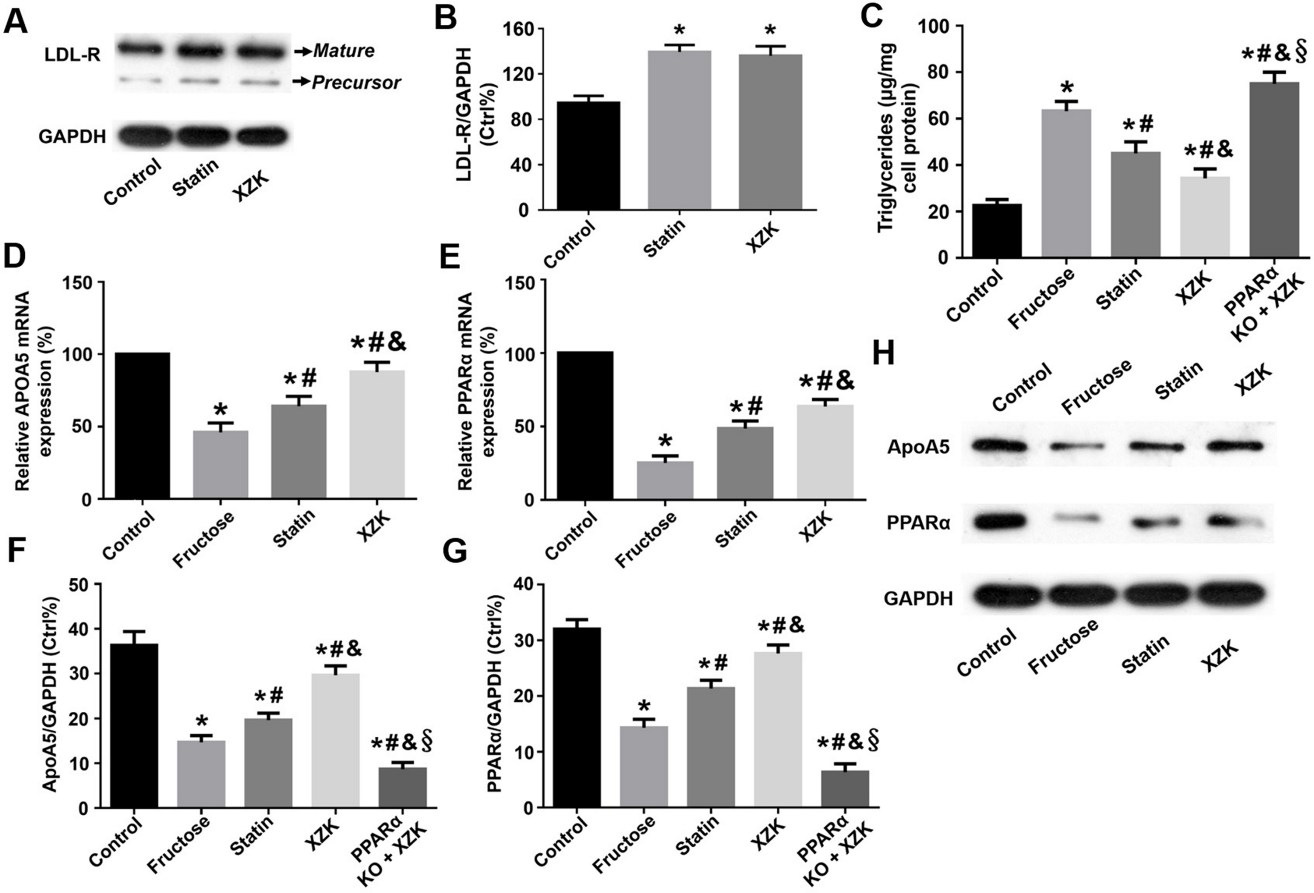
LDL-R, TG, apoA5 and PPARα in HepG2 cells. (A-B) LDL-R protein expressions: Two protein bands of LDL-R were exhibited including its precursor (120 kDa) and mature (160 kDa) forms. No significant difference of LDL-R expressions was indicated between the two treatments. (C) TG contents: The two agents effectively ameliorated fructose-induced TG elevation but this effect was more considerable in XZK group. Interestingly, PPARα down-regulation by shRNA effectively inhibited XZK-induced hypotriglyceridemic actions. (D-H) mRNA and protein of ApoA5 and PPARα: The two agents remarkably ameliorated fructose-induced down-regulation of apoA5 and PPARα mRNA and protein, whereas these effects were more significant in XZK group than statin group. However, PPARα knockdown eliminated these above effects of XZK. *^, #, &, §^ Values were significantly different from control, fructose, statin and XZK group, respectively (P < 0.05). LDL-R, low density lipoprotein receptor; TG, triglyceride; ApoA5, apolipoprotein A5; PPARα, peroxisome proliferator-activated receptor α; XZK, Xuezhikang.

In order to titrate a target dose for an equivalent LDL-C lowering power of the two agents (XZK and simvastatin), hepatocyte LDL-R protein expressions were used as an index. As shown in **Fig 2A**, two protein bands of LDL-R by Western blot analysis were exhibited including its precursor (120 kDa) and mature (160 kDa) forms as described previously [19]. No significant difference of LDL-R expressions was detected between the two treatments (XZK 500 μg/ml versus simvastatin 10 μg/ml, P > 0.05), suggesting the two treatments shared an equivalent power of LDL-R up-regulation. Therefore, it is plausible to compare their TG-lowering actions on hepatocytes.

Our study demonstrated an elevation of cellular TG contents in hepatocytes with treatment of fructose (P < 0.01). On the contrary, both XZK (500 μg/ml) and simvastatin (10 μg/ml) effectively attenuated fructose-induced TG elevation (both P < 0.05), and this effect was more considerable in XZK group than statin group (P < 0.05). However, PPARα knockdown using shRNA dramatically inhibited XZK-induced reduction of hepatocyte TG contents (P < 0.05) **(Fig 2B)**.

Furthermore, we compared the impact of the two treatments on hepatocyte apoA5 and PPARα expressions. Firstly, a down-regulation of apoA5 mRNA and protein was obtained in fructose group compared to control group (both P < 0.05). When treated with the two agents, fructose-induced apoA5 down-regulation was apparently ameliorated (all P < 0.05), and this effect was more remarkable in XZK group than statin group (P < 0.05). Interestingly, PPARα down-regulation by shRNA effectively repressed apoA5 up-regulation by XZK (P < 0.05) **(Fig 2C and 2D)**. Secondly, hepatocyte PPARα expressions were also markedly inhibited when administrated with fructose (both P < 0.05). By contrast, the two agents effectively reversed fructose-induced PPARα down-regulation, whereas this effect was more considerable in XZK group than statin group (all P < 0.05). As expected, PPARα knockdown remarkably inhibited XZK-induced PPARα up-regulation **(Fig 2E and 2F)**.

## Discussion

Hypertriglyceridemia represents an independent risk of CHD [2, 20], and the proatherogenic role of TG is associated with two other lipid disorders, including the formation of small, dense LDL and the reduction of HDL-C [21]. Clinically, it is not rare that plasma TG levels still remain high in some CHD patients when their LDL-C is satisfactorily controlled by statins [3, 4]. To address this issue, two strategies were recommended: upgrading the intensity of statin and/or adding a specific hypotriglyceridemic agent (*i*.*e*. combination lipid-lowering therapy) [20]. However, several concerns discourage their clinical application. Firstly, since the statins are an inhibitor of HMG-CoA reductase (a rate-limiting enzyme for hepatic cholesterol synthesis), the major benefit of upgrading the statin dose is to further lower LDL-C rather than TG. Instead, it will increase adverse effects of statins [1]. It is noteworthy that, owing to high incidence of statin-intolerance, high-intensity statin therapy is not reasonable for most Chinese CHD patients [22]. Secondly, although combination lipid-lowering therapy is alternatively recommended, it will inevitably increase the cost of medications and the risk of drug-drug interactions [1]. By contrast, XZK could be a superior alternative for these CHD patients.

Although the LDL-C lowering performance of XZK is modest (only equivalent to a low-intensity statin) [1, 4], the excellent hypotriglyceridemic potency of this agent has been confirmed [3, 4]. To date, however, no studies have been designed to compare the hypotriglyceridemic effects of XZK and statin based on an equivalent LDL-C lowering density. Because XZK is a multi-component polypill [4], it is unlikely to attribute the whole lipid-lowering effect of this agent exclusively to its statin components. Besides, the major purpose of this study was to investigate TG (not LDL-C)-lowering effects of the two agents. To address this issue, we adopted a tactic of titrating their target dose for an equivalent LDL-C lowering density. In our animal study, we titrated their doses (XZK 80mg/kg/d *versus* simvastatin 1mg/kg/d) according to their anticipated LDL-C reduction. As a result, the two treatments predicted an equivalent LDL-C reduction, revealing the two treatments shared an equivalent LDL-C lowering power. Interestingly, compared with simvastatin (1 mg/kg/d), XZK (80 mg/kg/d) more effectively reduces TG of these hypertriglyceridemia rats. Furthermore, we compared their hypotriglyceridemic effects of the two agents on hepatocytes *in vitro*. Considering the fact that LDL-C measurement cannot be obtained in hepatocytes *in vitro* and LDL-R on hepatocytes plays a key role in the LDL-C lowering effects of statins [17], hepatocyte LDL-R expressions were used as an index to titrate the target doses of the two agents (XZK 500 μg/ml *versus* simvastatin 10 μM) for an equivalent LDL-C lowering power *in vitro*. As a result, XZK (500 μg/ml) and simvastatin (10 μM) shared an equivalent LDL-R lowering power, whereas XZK (500 μg/ml) more effectively decreases hepatocyte TG contents than simvastatin (10 μM). All together, our findings *in vivo* and *in vitro* demonstrated that XZK, based on an equivalent LDL-C/LDL-R lowering power, has a better performance in TG reduction than simvastatin.

Considering the well-documented role of apoA5 in TG metabolism [8, 9, 23, 24] and fibrate/statin-related hypotriglyceridemic effects [14–16], we investigated the potential role of apoA5 in XZK-related hypotriglyceridemic actions. Our animal study demonstrated that XZK (80 mg/kg/d) contributes to more elevation of plasma apoA5 levels of rats than simvastatin (1 mg/kg/d), with an established inverse correlation between apoA5 and TG. More up-regulation of hepatic apoA5 expressions is identified in rats by XZK (80 mg/kg/d) than by simvastatin (1 mg/kg/d). Similarly, XZK (500 μg/ml) results in higher apoA5 expressions in hepatocytes *in vitro* than simvastatin (10 μM). Given apoA5 as a liver-specific protein [8, 9], we conclude that XZK increases hepatic apoA5 synthesis and therefore plasma apoA5 levels. Therefore, our study, on the one hand, supports the role of apoA5 in XZK-related hypotriglyceridemic actions. On the other hand, more apoA5 up-regulation is responsible for more TG reduction by XZK than simvastatin.

To date, three metabolic pathways have been implicated in apoA5-induced TG reduction: (1) inhibition on VLDL production and secretion [23], (2) stimulation of LPL-mediated TG hydrolysis [24], and (3) acceleration of hepatic uptake of VLDL and their remnants [25]. Interestingly, statin-related hypotriglyceridemic actions share the above three pathways [26]. Given that statins are the major components of XZK [4], it is expected that XZK-related lipid-lowering effects mainly result from its statin components. Actually, the role of apoA5 in statin-related hypotriglyceridemic actions has been identified by other and our previous studies [15, 16]. Thus, it is likely that statin components mainly contribute to XZK-induced TG reduction via the apoA5 pathway.

However, it is not reasonable to attribute the whole hypotriglyceridemic effect of this agent to its statin components. As a multi-component polypill, XZK has several non-statin hypotriglyceridemic components (for example, unsaturated fatty acids) [4]. On the other hand, the multi-component mode of XZK, an important feature of derivatives from traditional Chinese medicine [27], leads to a limitation of this study in that it is an immense challenge to precisely identify the individual pharmacological contributions of each active component of this agent. Unlike the current pharmacy perspective “one target, one drug”, multi-component mode has been regarded as a unique advantage of traditional Chinese medicine because the crosstalk between each active ingredient will produce a “1+1>2” synergistic action [28]. Therefore, it is likely that the multi-component mode of XZK, compared with the single-component mode of simvastatin, would produce more hypotriglyceridemic benefits.

Considering APOA5 as a target gene of PPAR-α [14], the potential role of PPAR-α in XZK-related hypotriglyceridemic effects was also investigated. Our previous study has confirmed the involvement of PPAR-α in statin-induced elevation of apoA5 and subsequently reduction of TG [16]. Similarly, this study demonstrated that simvastatin up-regulates apoA5 via the PPAR-α pathway that leads to a decrease of TG. More importantly, the role of PPAR-α was identified in XZK-related hypotriglyceridemic effects. In this study, XZK treatment contributes to up-regulation of hepatic PPARα expressions that is accompanied with apoA5 elevation and therefore TG reduction. However, PPARα down-regulation by shRNA almost eliminated the above effects of XZK on hepatocyte apoA5 and TG. Furthermore, we found out that XZK, based on an equivalent LDL-C/LDL-R lowering power, more effectively increases hepatic PPARα expressions than simvastatin. The multi-component mode of this agent may be responsible for this finding, because its several active components, such as statins [16, 29] and unsaturated fatty acids [30], have been shown to enhance PPARα expressions.

In summary, our study demonstrates that XZK has higher hypotriglyceridemic performance than simvastatin with an equivalent LDL-C lowering power, which supports XZK as a superior choice for CHD patients with modestly increased LDL-C but markedly elevated TG, wherein more hepatic apoA5 synthesis by this agent is involved via the signaling PPARα pathway. Theoretically, these pharmacological benefits of this agent could be associated with its unique multi-component mode.
